# Cadaveric Study Reveals Anatomical Variations of the Recurrent Thenar Branch of the Median Nerve in the Carpal Tunnel

**DOI:** 10.7759/cureus.89334

**Published:** 2025-08-04

**Authors:** Aga Ammar Murthuza, Surendra Babu T, Betty Ana Jose, Vinay Srinivas, Varsha Mokhasi, Venkata BharatKumar Pinnelli, Venkataramana Kandi

**Affiliations:** 1 Anatomy, Vydehi Institute of Medical Sciences and Research Centre, Bangalore, IND; 2 Biochemistry, Vydehi Institute of Medical Sciences and Research Centre, Bangalore, IND; 3 Clinical Microbiology, Prathima Institute of Medical Sciences, Karimnagar, IND

**Keywords:** carpal tunnel release, carpal tunnel syndrome, flexor retinaculum, median nerve, recurrent thenar branch

## Abstract

Background

Carpal tunnel syndrome (CTS) is one of the most prevalent types of entrapment mononeuropathies, necessitating surgical treatment. The median nerve and its branches within the carpal tunnel have anatomical variances that may have clinical implications due to the possibility of iatrogenic injury while undergoing decompression treatments.

Methods

A total of 40 upper limb specimens (17 right and 23 left) from the Department of Anatomy were used in the dissection investigation. The recurrent thenar branch (RTB) was discovered and identified based on Lanz's classification system. Four characteristics were measured using calipers: flexor retinaculum (FR) width, distances between the superficial palmar arch (SPA) by measuring from the distal border of the FR and the RTB origin, the distance between the proximal border of the FR and the palmar cutaneous branch (PCB) of the median nerve, and the distance between the distal border of the FR and the origin of the RTB of the median nerve. Statistical tests were used to compare the left and right sides, with chi-square tests (χ^2^) analyzing Lanz's classification distribution.

Results

Of the 40 specimens examined, in 42.5% (n=17), RTB of the median nerve had a subligamentous course (Type 1A), 32.5% (n=13) had an extraligamentous course (Type 0), and 20% (n=8) had a transligamentous course (Type 1B). One (2.5%) specimen had an ulnar variation (Type 1C), while another had an accessory branch (Type 4A). The anatomical relationships of the RTB and accompanying tissues are consistent throughout both sides of the body in the cases examined (p>0.05).

Conclusions

This study demonstrated significant anatomical variations of the median nerve in the carpal tunnel with a focus on the RTB. The high incidence of transligamentous RTB emphasizes the need for meticulous surgical planning during carpal tunnel release procedures to avoid nerve injury.

## Introduction

Carpal tunnel syndrome (CTS) is a common condition in which the median nerve is compressed as it travels through the carpal tunnel in the wrist. CTS affects approximately 3% of the adult population, primarily women and elderly individuals. Repetitive hand movements, obesity, and certain chronic conditions such as rheumatoid arthritis are all common risk factors for the disorder [1]. Individuals with CTS may experience symptoms such as hand pain, numbness, and weakness, particularly in the thumb, index, middle fingers, and the lateral half of the ring finger [1,2]. Surgical intervention, specifically carpal tunnel release (CTR), is the treatment for CTS, with over 500,000 procedures performed yearly in the United States [1,3]. Although the technique appears easy, it is not without hazards. One of the most serious concerns is the iatrogenic damage to the median nerve's recurrent thenar branch (RTB), which may result in functional impairments of the hands. The RTB of the median nerve, arising typically from the median nerve distal to the flexor retinaculum within the carpal tunnel, supplies motor innervation to the intrinsic thenar muscles, including the abductor pollicis brevis, flexor pollicis brevis, and opponens pollicis. The RTB innervates the thenar muscles, which are important for thumb opposition and grip strength [1,4]. Anatomical differences in the median nerve and its branches, particularly the RTB, have been widely characterized and can impact surgical results [1,3,5]. Anatomically, the RTB of the median nerve arises from the median nerve within the carpal tunnel and courses distally toward the thenar muscles, with its pathway varying depending on the FR. Variations in the thenar motor branch (TMB) course can be identified using Lanz's classification, which provides a detailed framework for identifying and categorizing anatomical variations in the course and branching patterns of the RTB of the median nerve within the carpal tunnel. According to Lanz's classification, the RTB of the median nerve exhibits a wide spectrum of anatomical variations, including subligamentous, transligamentous, ulnarward, supraligamentous, and extraligamentous courses, while also identifying accessory branches that may arise proximally or distally, traversing directly through the thenar musculature or joining with other neural branches and describing the high division of the median nerve, which may be accompanied by a concomitant artery or the presence of a lumbrical muscle [1,4-6].

Understanding these variances is crucial for surgeons in reducing the risk of nerve injury during CTR procedures [1,3]. Previous research has found a wide variety of incidence rates for various RTB variants, with extraligamentous courses being the most prevalent [3,7]. Individuals may have dramatically different anatomical connections between the RTB, the FR, and the superficial palmar arch (SPA) [8]. This heterogeneity necessitates detailed anatomical knowledge and meticulous surgical technique in avoiding problems [1,3].

The current study evaluated the anatomical differences of the RTB of the median nerve in a sample of 40 upper limb specimens. This study employed Lanz's classification to measure critical anatomical features to improve understanding of RTB variations and their implications for surgical treatment. The findings will contribute to the existing body of information and may lead to safer surgical approaches for CTR, ultimately enhancing patient outcomes.

## Materials and methods

Study design and specimens

This anatomical study used 40 upper limb specimens, 17 right and 23 left hands, obtained from the Department of Anatomy at the Vydehi Institute of Medical Sciences and Research Centre (VIMS & RC), Bangalore, Karnataka, India. A convenience sampling method was employed, including all suitable upper limb specimens available during the study period, without any gender-specific selection. The specimens were selected based on their appropriateness for dissection, with no gross anatomical defects or prior surgical procedures that could influence the anatomical relationships of the median nerve and its branches. The investigation was conducted in alignment with ethical regulations and guidelines for anatomical research, ensuring that all specimens were handled with respect and that the study adhered to institutional regulations regarding the use of human cadaveric material. The study was approved by the Institutional Ethics Committee of VIMS & RC (Ref. No. VIEC/2025/APP/15).

Dissection procedure

The dissection was done using traditional anatomical procedures. On the palmar aspect of each hand, a midline incision was made from the distal wrist crease to the proximal interphalangeal joint of the ring finger, and the dissection procedure was performed with standard anatomical procedures. The skin and subcutaneous layer were meticulously dissected to reveal the underlying anatomy, which included the FR, median nerve, and its branches.

Identification and classification of the RTB

The RTB of the median nerve was identified during the dissection. The classification of the RTB was performed based on Lanz's classification system, which categorizes the branch into the following types: Type 0: extraligamentous type, which has the RTB passing outside the FR; Type 1A: subligamentous type, which passes beneath the ligament; Type 1B: transligamentous type, which pierces through the ligament; Type 1C: the ulnar variant arises from the ulnar side of the median nerve; and Type 4A: accessory branch, which indicates the presence of an additional branch [4-6].

Measurement parameters

**Figure 1:**
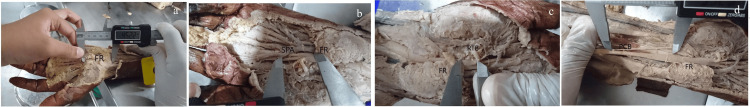
Anatomical illustration and cadaver dissection of the hand (a) The width was measured at the midpoint of the FR; (b) the distance between the lowermost part of the SPA and the distal border of the FR; (c) the distance between the origin of the RTB and the distal border of the FR; (d) the distance between the origin of the PCB and the proximal border of the FR. FR: flexor retinaculum; SPA: superficial palmar arch; RTB: recurrent thenar branch; PCB: palmar cutaneous branch. Image credit: The authors

Four critical anatomical parameters were evaluated using vernier calipers (Corceptive Infrastructure Private Limited, Nagpur, India), which were calibrated to measure 0-6 in/0-150 mm, featuring an inch-to-millimeter conversion, an extra-large liquid crystal display, and an auto-off feature. These calipers were used to assess the relationships between the RTB and its surroundings. The width of the FR was measured at its midpoint, from the most prominent point of the SPA at the level of the hook of hamate to the distal border of the FR, from the origin of the RTB to the distal border of the FR, and from the origin of the palmar cutaneous branch (PCB) to the proximal border of the FR (Figure 1).

Statistical analysis

The collected data were analyzed to determine the prevalence of each form of RTB according to Lanz's classification. The tables were prepared using Microsoft Office 2019 Excel (Microsoft Corp., Redmond, WA, USA). IBM SPSS Statistics for Windows, Version 20 (Released 2011; IBM Corp., Armonk, New York) software was utilized to conduct statistical analysis and calculate mean values and standard deviations for all anatomical data observed. Side-related differences between the right and left hands were explored, and chi-square tests were employed to establish the distribution of morphological variants classified by Lanz. A p-value < 0.05 indicated statistical significance.

## Results

Distribution of RTB variations according to Lanz's classification

The anatomical differences of the RTB of the median nerve in the 40 specimens investigated were classified using Lanz's categorization. The most common variety found was the subligamentous type (17, 42.50%), followed by the extraligamentous type (13, 32.50%) and the transligamentous type (08, 20%). Only one specimen (2.5%) had the ulnar variation or the accessory branch types recognized. The distribution of these variants is shown in Table 1.

**Table 1:** Distribution of recurrent thenar branch variations according to Lanz's classification Data have been represented as N%, and the p-value was derived from the chi-square value. A p-value < 0.05 is considered significant. NA: not applicable. This table has been created by the authors.

Lanz's Classification	Right (n=17) (N%)	Left (n=23) (N%)	Total (n=40) (N%)	Chi-square Value (χ^2^)	p-value
Extraligamentous (0)	04 (23.50)	09 (39.13)	13 (32.50)	1.085	0.896
Subligamentous (1A)	08 (47.10)	09 (39.13)	17 (42.50)	0.251	0.992
Transligamentous (1B)	04 (23.50)	04 (17.39)	08 (20)	0.226	0.994
Ulnar variant (1C)	00 (00)	01 (04.35)	01 (02.50)	NA	NA
Accessory branch (4A)	01 (05.90)	00 (00)	01 (02.50)	NA	NA

Measurements of anatomical parameters

Various anatomical parameters were measured on both the right and left hands. The FR width, the distance between the lowermost part of the SPA and the distal border of the FR, the distance between the proximal border of the FR and the origin of PCB of the median nerve, and the distance between the distal border of the FR and the origin of RTB of the median nerve were all measured. The statistical analysis revealed no significant difference in measured values between the right and left hands (p > 0.05). This indicates that the anatomical relationships of the RTB and accompanying tissues are consistent throughout both sides of the body in the cases examined, as shown in Table 2.

**Table 2:** Measurements of anatomical parameters The data are represented as mean ± SD, and the p-value was calculated using a two-tailed t-test. A p-value < 0.05 is considered significant. FR: flexor retinaculum; SPA: superficial palmar arch; PCB: palmar cutaneous branch of median nerve; RTB: recurrent branch of median nerve; mm: millimeters; SD: standard deviation. This table has been created by the authors.

Measurement (Units)	Right Hand (n=17) (Mean±SD)	Left Hand (n=23) (Mean±SD)	t-value	p-value
Width of FR (mm)	32.97±08.04	35.90±09.05	0.864	0.393
Distance between FR (distal border) and SPA (mm)	15.59±09.39	17.99±12.17	0.677	0.502
Distance between FR (proximal border) and PCB (mm)	36.06±12.43	32.17±13.27	-0.941	0.352
Distance between FR (distal border) and RTB (mm)	06.02±04.05	05.05±03.16	-0.851	0.399

## Discussion

The RTB of the median nerve is critical to the motor function of the thenar muscles, which are required for thumb opposition and grip strength. Awareness of the anatomical variability of the RTB is essential to reduce the risk of iatrogenic injury during surgical procedures such as CTR. The purpose of this study was to classify RTB variants using Lanz's categorization and to measure critical anatomical features in 40 upper limb specimens. The current study found that the most common variation of the RTB was the subligamentous type (17, 42.50%), followed by the extraligamentous type (13, 32.50%) and the transligamentous type (08, 20%). Only one (2.5%) of the specimens contained the ulnar variant or the accessory branch type. These findings are consistent with prior research, which found variable prevalence rates for these sorts of polymorphisms. For example, a systematic review by Henry et al. found that the extraligamentous variety is frequently the most common, with rates ranging from 55% to 96% across studies [7]. These variations might have been influenced by the sample sizes of the respective studies. The anatomical differences of the RTB can have a major impact on surgery outcomes. For example, the extraligamentous path offers a safer approach during CTR. This is because the nerve is positioned distal to the transverse carpal ligament (TCL) and is less prone to sustaining injuries during the treatment. In contrast, the transligamentous route increases the chance of damage because the nerve penetrates the TCL [9]. The TCL and the FR are different terms for the same structure that forms the roof of the carpal tunnel.

These changes have far-reaching clinical ramifications. Damage to the RTB during CTR can result in significant functional deficiencies, such as weak thumb opposition and grip strength, which can impair a patient's quality of life. The RTB's "million-dollar nerve" moniker emphasizes the necessity of maintaining this nerve during surgical procedures [3,9]. Surgeons should consider anatomical variations during the CTR planning and execution phases. Intraoperative imaging has been proposed as a tool for visualizing RTB and its progression, particularly in cases where anatomical changes are suspected. This technique may increase the procedure's safety by allowing real-time tracking of the nerve's position relative to the TCL [3,10].

This study not only classified the differences but also examined various anatomical characteristics, such as the width of the FR and the distances between major anatomical landmarks. The results showed that there were no statistically significant differences between the right and left hands for any of the characteristics tested (p > 0.05). This study implies that the anatomical linkages of the RTB and accompanying tissues are consistent on both sides of the body, which is useful for surgical planning. Surgeons might benefit from knowing the average width of the FR as well as the distances measured from it to the SPA, PCB, and RTB of the median nerve.

For example, knowing the typical distance between the FR's distal border and the RTB of the median nerve can help surgeons avoid injuring the nerve during dissection [10,11]. The current study highlights the occurrence of numerous anatomical variants of the RTB of the median nerve, emphasizing the significance of recognizing these variations for safer surgical methods. The findings suggest that during CTR, meticulous inspection and consideration of anatomical components are required to reduce the risk of nerve injury. As surgical techniques advance, new imaging modalities, including magnetic resonance imaging (MRI), can be used to characterize the architecture of the RTB in vivo, which may help to improve the safety and efficacy of surgeries involving the median nerve and its branches [2,8,12].

Study limitations

While the current study provides valuable insights into the structural differences of the RTB, it has limitations. The sample size of 40 specimens without gender-specific analysis may not accurately reflect the diversity of anatomical differences found in the general population. Furthermore, inter-rater and intra-rater reliability analyses were not performed in this study. Future studies with larger sample numbers and other demographic groups are needed to confirm these findings and explore the therapeutic significance of the observed discrepancies. The study's use of cadaveric specimens may not account for the dynamic changes that occur in living individuals.

## Conclusions

The current study assessed the key anatomical variances of the median nerve's RTB, emphasizing the importance of recognizing these variations for safer surgical procedures, particularly during CTR. According to the findings, the most common variation is a subligamentous path, followed by extraligamentous and transligamentous varieties. The presence of these variations raises the risk of iatrogenic injury during surgical procedures. Therefore, surgeons must be aware of any anatomical complexities they may encounter. The data acquired on anatomical factors, such as the width of FR and distances to critical landmarks, are useful for surgical planning. The lack of statistically significant differences between the right and left hands suggests that these anatomical linkages are stable on both sides, which can help with preoperative assessments and intraoperative decision-making.

Given the high incidences of anatomical differences and their consequences for surgical outcomes, incorporating modern imaging techniques, such as MRI, may improve the detection of these variations before surgery. This method has the potential to provide more precise surgical treatments while also lowering the likelihood of CTR complications. Reducing the risk of nerve damage and enhancing surgical outcomes requires a decent grasp of the median nerve and its branches. Future research should examine these differences, their prevalence, and clinical significance, as well as the possible benefits of integrating imaging methods into routine surgical procedures.
